# Supplementary material for: Drug repurposing for aging research using model organisms
Source: Aging Cell. 2017 Jun 16;16(5):1006–15. doi: 10.1111/acel.12626 (PMC5595691; doi:10.1111/acel.12626)
Supplement: Supplementary file 7 — Data S1 Zip‐Archive of all report cards. [file ACEL-16-1006-s007.zip › RC_DMS.pdf]

DMS

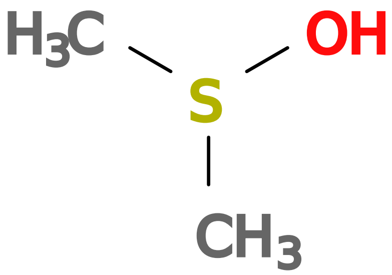

Database identifiers

ChEMBLCompound	CHEMBL504
DrugBank	DB01093
CHEBI	28262
ZINC	ZINC05224188
eMolecules	483582

Ranking

	Rank	Score
Drosophila	480/697	0.295
C. elegans	480/591	0.092

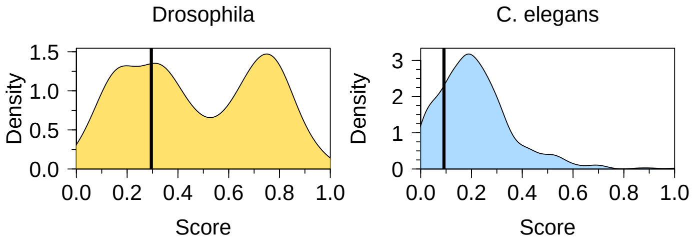

	Ageing implication	Domain conservation	Binding site conservation	Binding affinity	Bioavailability	Lipinski	Promiscuity	Purchasability	Drug approval	**Total**
Drosophila	1.0	0.973	1.0	0.336	(0.9)	0.0	-0.199	0.1	0.1	0.295
C. elegans	1.0	0.97	1.0	0.336	0.278	0.0	-0.199	0.1	0.1	0.092

Names

- **dimethyl sulfoxide**
- (ch3)2so
- dimethyl sulfur oxide
- DMSO
- Infiltrina
- methylsulfinylmethane
- Rimso 50
- S(O)Me2
- sulfinylbis(methane)
- Syntexan

Roles

polar aprotic solvent, radical scavenger, non-narcotic analgesic, antidote, MRI contrast agent, alkylating agent

Status

Approved drug (according to ChEMBL)	Yes
Administration Route	Intravesical, Topical
Number of Rule of 5 violations	0
Binding affinity to original target in log units (RF-Score prediction)	4.32
Burns *C. elegans* bioavailability prediction	-1.02

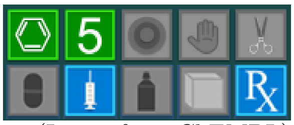

(Image from ChEMBL)

Medical Information

Indicatation: For the symptomatic relief of patients with interstitial cystitis.
Mechanism of action: The mechanism of dimethyl sulfoxide's actions is not well understood. Dimethyl sulfoxide has demonstrated antioxidant activity in certain biological settings. For example, the cardiovascular protective effect of dimethyl sulfoxide in copper-deficient rats is thought to occur by an antioxidant mechanism. It is also thought that dimethyl sulfoxide's possible anti-inflammatory activity is due to antioxidant action.
Toxicity: The oral LD50 of dimethyl sulfoxide in the dog is greater than 10 gm/kg. It is improbable that this dosage level could be obtained with intravesical instillation of dimethyl sulfoxide in the patient.
Metabolism: Dimethyl sulfoxide is metabolized in man by oxidation to dimethyl sulfone or by reduction in dimethyl sulfide. Dimethyl sulfoxide and dimethyl sulfone are excreted in the urine and feces.
Retail: Ben Venue Laboratories Inc., Bioniche Pharma, Bryan Corp., Edwards Lifesciences LLC, Medisca Inc.
Dimethyl Sulfoxide Irrigation 50 % Solution - 1.05 USD / ml
Rimso-50 solution - 1.15 USD / ml
Rimso-50 50 % Solution - 1.25 USD / ml
Sclerosol intrapleural aero - 3.98 USD / g
Rimso-50 50% Solution 50ml Vial - 111.2 USD / vial
Kemsol 70 % Solution - 0.31 USD / ml
and 2 more options

(Information from DrugBank)

Compound Target Characteristics

Menin

Best gene implication in ageing for this target family came from gene Q8SXU2 annotated in UniProt release 2014_02. Annotation GO 8340 (determination of adult lifespan) was Inferred from Mutant Phenotype

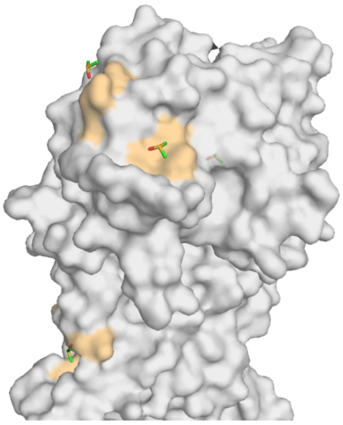

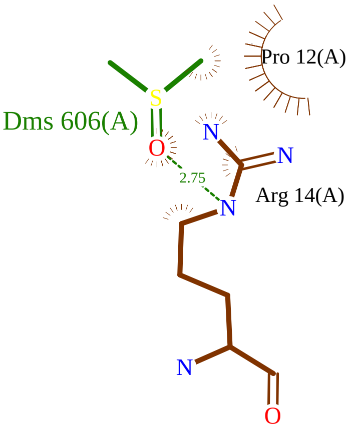

protein	amino acids contacts (binding site)
PDB:4gq4:chainA:O00255	P R F F P V V G E Y R R N V R S A S
PDB:4og3:chainA:O00255	P R F F P V V G E Y R R N V R S A S
PDB:4og4:chainA:O00255	P R F F P V V G E Y R R N V R S A S
PDB:4og5:chainA:O00255	P R F F P V V G E Y R R N V R S A S
PDB:4og6:chainA:O00255	P R F F P V V G E Y R R N V R S A S
PDB:4og7:chainA:O00255	P R F F P V V G E Y R R N V R S A S
PDB:4og8:chainA:O00255	P R F F P V V G E Y R R N V R S A S
sp:O00255:MEN1_HUMAN	P R F F P V V G E Y R R N V R S A S
sp:Q9WVR8:MEN1_RAT	P R F F P V V G E Y R R N V R S A S
sp:O88559:MEN1_MOUSE	P R F F P V V G E Y R R N V R S A S
tr:F8WHD9:F8WHD9_MOUSE	P R F F P V V G E Y R R N V R S A S
tr:Q8SXU2:Q8SXU2_DROME	P K Y S P V V R V A V L K Y R S I S
tr:Q7KXY9:Q7KXY9_DROME	P K Y S P V V R V A V L K Y R S I S
tr:Q9VM47:Q9VM47_DROME	P K Y S P V V R V A V L K Y R S I S

	whole protein		domain-based		contact-based	
protein	ident	simil	ident	simil	ident	simil
PDB:4gq4:chainA:O00255	1.0	1.0	1.0	1.0	1.0	1.0
PDB:4og3:chainA:O00255	1.0	1.0	1.0	1.0	1.0	1.0
PDB:4og4:chainA:O00255	1.0	1.0	1.0	1.0	1.0	1.0
PDB:4og5:chainA:O00255	1.0	1.0	1.0	1.0	1.0	1.0
PDB:4og6:chainA:O00255	1.0	1.0	1.0	1.0	1.0	1.0
PDB:4og7:chainA:O00255	1.0	1.0	1.0	1.0	1.0	1.0
PDB:4og8:chainA:O00255	1.0	1.0	1.0	1.0	1.0	1.0
sp:O00255:MEN1_HUMAN	1.0	1.0	1.0	1.0	1.0	1.0
sp:Q9WVR8:MEN1_RAT	0.96	0.98	0.96	0.98	1.0	1.0
sp:O88559:MEN1_MOUSE	0.96	0.98	0.96	0.98	1.0	1.0
tr:F8WHD9:F8WHD9_MOUSE	0.95	0.97	0.95	0.97	1.0	1.0
tr:Q8SXU2:Q8SXU2_DROME	0.36	0.64	0.37	0.65	0.39	0.51
tr:Q7KXY9:Q7KXY9_DROME	0.32	0.6	0.34	0.64	0.39	0.51
tr:Q9VM47:Q9VM47_DROME	0.32	0.59	0.34	0.64	0.39	0.51

Mnn1 (FBgn0031885) associated phenotypes

DNA repair defective, chemical sensitive, conditional, heat sensitive, lethal - all die before end of P-stage, melanotic mass phenotype, radiation sensitive, short lived, some die during pharate adult stage, stress response defective

(Information from FlyBase)

3-phosphoinositide-dependent protein kinase 1

Best gene implication in ageing for this target family came from gene Q9Y1J3 via mapping the annotation from Ensembl WBGene00003965 via mapping the annotation from EntrezGene 180475 via mapping the annotation from GenAgeModels 0499 annotated in GenAge release 17.

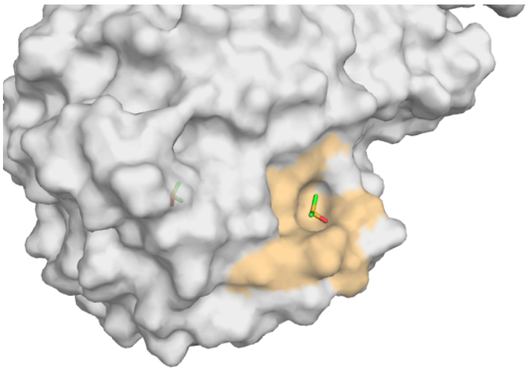

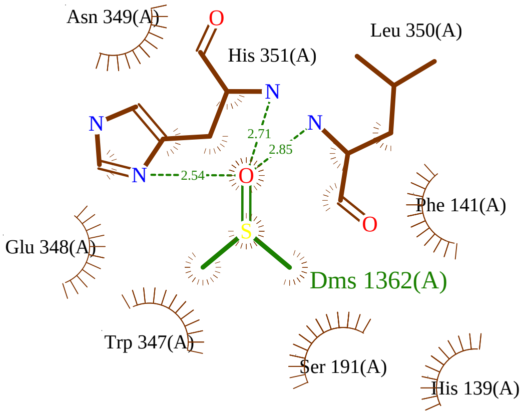

protein	amino acids contacts (binding site)											
PDB:4a06:chainA:O15530	H	P	F	R	G	S	G	W	E	N	L	H
PDB:4aw0:chainA:O15530	H	P	F	R	G	S	G	W	E	N	L	H
PDB:4aw1:chainA:O15530	H	P	F	R	G	S	G	W	E	N	L	H
PDB:4ct1:chainA:O15530	H	P	F	R	G	S	G	W	E	N	L	H
tr:Q9UPJ8:Q9UPJ8_HUMAN	H	P	F	R	G	S	G	W	E	N	L	H
tr:E9PER6:E9PER6_HUMAN	H	P	F	R	G	S	G	W	E	N	L	H
tr:C9JWR9:C9JWR9_HUMAN	H	P	F	R	G	S	G	W	E	N	L	H
sp:O15530:PDPK1_HUMAN	H	P	F	R	G	S	G	W	E	N	L	H
sp:O55173:PDPK1_RAT	H	P	F	R	G	S	G	W	E	N	L	H
tr:Q3UEW8:Q3UEW8_MOUSE	H	P	F	R	G	S	G	W	E	N	L	H
tr:F2Z400:F2Z400_MOUSE	H	P	F	R	G	S	G	W	E	N	L	H
tr:Q3TRL2:Q3TRL2_MOUSE	H	P	F	R	G	S	G	W	E	N	L	H
tr:F2Z3X6:F2Z3X6_MOUSE	H	P	F	R	G	S	G	W	E	N	L	H
sp:Q9Z2A0:PDPK1_MOUSE	H	P	F	R	G	S	G	W	E	N	L	H
tr:Q810Z4:Q810Z4_MOUSE	H	P	F	R	G	S	G	W	E	N	L	H
tr:A4V166:A4V166_DROME	N	P	G	N	G	L	G	W	Q	T	L	R
tr:H1ZY86:H1ZY86_DROME	N	P	G	N	G	L	G	W	Q	T	L	R
tr:H1ZY83:H1ZY83_DROME	N	P	G	N	G	L	G	W	Q	T	L	R
tr:A4V164:A4V164_DROME	N	P	G	N	G	L	G	W	Q	T	L	R
tr:C7LAC8:C7LAC8_DROME	N	P	G	N	G	L	G	W	Q	T	L	R
tr:H5V8B9:H5V8B9_DROME	N	P	G	N	G	L	G	W	Q	T	L	R
sp:Q9W0V1:PDPK1_DROME	N	P	G	N	G	L	G	W	Q	T	L	R
sp:Q9Y1J3:PDPK1_CAEEL	Q	P	F	C	G	T	G	W	V	N	I	A
sp:Q03407:PKH1_YEAST	G	K	G	K	G	D	G	F	E	D	V	W
sp:Q03306:PKH3_YEAST	Q	A	G	H	G	D	G	W	N	D	I	W
sp:Q12236:PKH2_YEAST	N	P	S	K	G	D	G	F	K	D	V	W

protein	whole protein ident	simil	domain-based ident	simil	contact-based ident	simil
PDB:4a06:chainA:O15530	0.99	1.0	0.99	0.99	1.0	1.0
PDB:4aw0:chainA:O15530	0.99	0.99	0.99	0.99	1.0	1.0
PDB:4aw1:chainA:O15530	0.99	1.0	0.99	0.99	1.0	1.0
PDB:4ct1:chainA:O15530	0.96	0.99	0.98	0.99	1.0	1.0
tr:Q9UPJ8:Q9UPJ8_HUMAN	0.66	0.66	1.0	1.0	1.0	1.0
tr:E9PER6:E9PER6_HUMAN	0.95	0.95	1.0	1.0	1.0	1.0
tr:C9JWR9:C9JWR9_HUMAN	0.95	0.96	0.88	0.89	1.0	1.0
sp:O15530:PDPK1_HUMAN	1.0	1.0	1.0	1.0	1.0	1.0
sp:O55173:PDPK1_RAT	0.95	0.98	0.99	1.0	1.0	1.0
tr:Q3UEW8:Q3UEW8_MOUSE	0.73	0.76	0.99	1.0	1.0	1.0
tr:F2Z400:F2Z400_MOUSE	0.75	0.79	0.99	1.0	1.0	1.0
tr:Q3TRL2:Q3TRL2_MOUSE	0.88	0.91	0.99	1.0	1.0	1.0
tr:F2Z3X6:F2Z3X6_MOUSE	0.9	0.93	0.99	1.0	1.0	1.0
sp:Q9Z2A0:PDPK1_MOUSE	0.95	0.98	0.99	1.0	1.0	1.0
tr:Q810Z4:Q810Z4_MOUSE	0.93	0.97	0.99	1.0	1.0	1.0
tr:A4V166:A4V166_DROME	0.3	0.49	0.41	0.65	0.42	0.58
tr:H1ZY86:H1ZY86_DROME	0.3	0.5	0.42	0.65	0.42	0.58
tr:H1ZY83:H1ZY83_DROME	0.3	0.5	0.42	0.65	0.42	0.58
tr:A4V164:A4V164_DROME	0.31	0.56	0.42	0.65	0.42	0.58
tr:C7LAC8:C7LAC8_DROME	0.28	0.5	0.42	0.65	0.42	0.58
tr:H5V8B9:H5V8B9_DROME	0.28	0.5	0.42	0.65	0.42	0.58
sp:Q9W0V1:PDPK1_DROME	0.28	0.5	0.42	0.65	0.42	0.58
sp:Q9Y1J3:PDPK1_CAEEL	0.28	0.6	0.34	0.66	0.5	0.63
sp:Q03407:PKH1_YEAST	0.21	0.47	0.44	0.72	0.25	0.56
sp:Q03306:PKH3_YEAST	0.15	0.36	0.38	0.68	0.25	0.67
sp:Q12236:PKH2_YEAST	0.15	0.35	0.44	0.73	0.25	0.61

Pdk1 (FBgn0020386) associated phenotypes

cell autonomous, chemical conditional, chemical resistant, chemical sensitive, decreased cell size, developmental rate defective, increased cell death, increased cell size, male limited, neuroanatomy defective, nutrition conditional, partially lethal - majority die, small body, somatic clone

(Information from FlyBase)

Pdk1 (UniProt:Q9W0V1) annotation

Function: Serine/threonine kinase required for embryonic development. Inhibits apoptosis. Acts in the insulin receptor transduction pathway which regulates cell growth and organ size, by phosphorylating and activating Akt1 and S6k. May be involved in axonal pathfinding and synaptogenesis, and in spermatogenesis. (PubMed:10336630, PubMed:11301252, PubMed:11344272, PubMed:11752451, PubMed:11862217, PubMed:15238523, PubMed:9368760).
Subcellular location: Cytoplasm
Developmental stage: Highly expressed in Malpighian tubule primordia from stage late 11 to 13. Expressed in hindgut at stage 13. After stage 14, ubiquitously expressed at low levels. (PubMed:11301252).
Domain: The PIF-pocket is a small lobe in the catalytic domain required by the enzyme for the binding to the hydrophobic motif of its substrates. It is an allosteric regulatory site that can accommodate small compounds acting as allosteric inhibitors. (UniProtKB:O15530).
Disruption phenotype: Death during the second instar stage. (PubMed:11752451).

(Information from UniProt)

pdk-1 (WBGene00003965) associated phenotypes

DMPP resistant, carbon dioxide avoidance variant, dauer constitutive, dauer recovery inhibited, egg laying variant, extended life span, long, reduced brood size, shortened life span, social feeding increased

(Information from WormBase)

pdk-1 (UniProt:Q9Y1J3) annotation

Function: Involved in the daf-2/insulin receptor-like transduction pathway, which controls longevity

and prevents developmental arrest at the dauer stage. Phosphorylates and activates sgk-1, akt-1 and akt-2. (PubMed:10364160, PubMed:15068796).
Subunit: Interacts directly with sgk-1, akt-1 and akt-2.(PubMed:15068796).
Subcellular location: Cytoplasm
Developmental stage: Expressed in late stage embryos and throughout life. At L1, expressed in neurons, intestinal cells and hypodermal cells. In adults, expressed in the somatic gonad. (PubMed:10364160).
Domain: The PIF-pocket is a small lobe in the catalytic domain required by the enzyme for the binding to the hydrophobic motif of its substrates. It is an allosteric regulatory site that can accommodate small compounds acting as allosteric inhibitors. (UniProtKB:O15530).

(Information from UniProt)

GTPase KRas

Best gene implication in ageing for this target family came from gene P01112 annotated in UniProt release 2014_02. Annotation GO subterm of 7568 (aging) was Inferred from Direct Assay

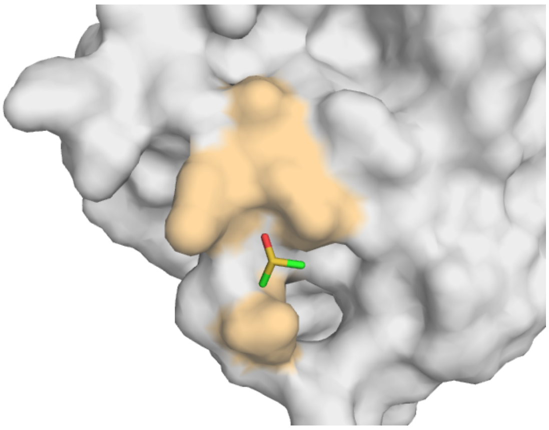

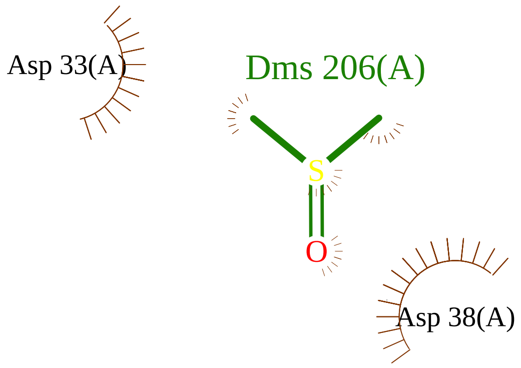

protein	amino acids contacts (binding site)
PDB:4dst:chainA:P01116	D I E D
tr:L7RSL8:L7RSL8_HUMAN	D I E D
sp:P01111:RASN_HUMAN	D I E D
tr:Q5U091:Q5U091_HUMAN	D I E D
sp:P01112:RASH_HUMAN	D I E D
sp:P01116:RASK_HUMAN	D I E D
tr:I1SRC5:I1SRC5_HUMAN	D I E D
tr:Q8CGQ3:Q8CGQ3_RAT	D I E D
sp:P20171:RASH_RAT	D I E D
sp:Q04970:RASN_RAT	D I E D
sp:P08644:RASK_RAT	D I E D
tr:A0JN17:A0JN17_RAT	D I E D
tr:Q71SW8:Q71SW8_MOUSE	D I E D
tr:P97870:P97870_MOUSE	D I E D
tr:C0H5X4:C0H5X4_MOUSE	D I E D
sp:Q61411:RASH_MOUSE	D I E D
sp:P32883:RASK_MOUSE	D I E D
tr:Q0VDV7:Q0VDV7_MOUSE	D I E D
tr:Q5J7N1:Q5J7N1_MOUSE	D I E D
tr:Q9D091:Q9D091_MOUSE	D I E D
tr:Q4FJP3:Q4FJP3_MOUSE	D I E D
sp:P08646:RAS1_DROME	D I E D
sp:P22981:LET60_CAEEL	D I E D
sp:P01119:RAS1_YEAST	D I E D
sp:P01120:RAS2_YEAST	D I E D

	whole protein		domain-based		contact-based	
protein	ident	simil	ident	simil	ident	simil
PDB:4dst:chainA:P01116	0.91	0.96	0.95	0.98	1.0	1.0
tr:L7RSL8:L7RSL8_HUMAN	1.0	1.0	1.0	1.0	1.0	1.0
sp:P01111:RASN_HUMAN	0.87	0.96	0.91	0.97	1.0	1.0
tr:Q5U091:Q5U091_HUMAN	0.87	0.96	0.91	0.97	1.0	1.0
sp:P01112:RASH_HUMAN	0.86	0.95	0.9	0.97	1.0	1.0
sp:P01116:RASK_HUMAN	1.0	1.0	1.0	1.0	1.0	1.0
tr:I1SRC5:I1SRC5_HUMAN	0.64	0.64	1.0	1.0	1.0	1.0
tr:Q8CGQ3:Q8CGQ3_RAT	0.51	0.51	0.55	0.55	1.0	1.0
sp:P20171:RASH_RAT	0.86	0.95	0.9	0.97	1.0	1.0
sp:Q04970:RASN_RAT	0.87	0.96	0.92	0.97	1.0	1.0
sp:P08644:RASK_RAT	0.99	1.0	0.99	1.0	1.0	1.0
tr:A0JN17:A0JN17_RAT	0.89	0.95	0.94	0.98	1.0	1.0
tr:Q71SW8:Q71SW8_MOUSE	0.5	0.51	0.54	0.55	1.0	1.0
tr:P97870:P97870_MOUSE	0.51	0.51	0.55	0.55	1.0	1.0
tr:C0H5X4:C0H5X4_MOUSE	0.52	0.58	0.56	0.63	1.0	1.0
sp:Q61411:RASH_MOUSE	0.86	0.95	0.9	0.97	1.0	1.0
sp:P32883:RASK_MOUSE	0.99	1.0	0.99	1.0	1.0	1.0
tr:Q0VDV7:Q0VDV7_MOUSE	0.99	1.0	0.99	1.0	1.0	1.0
tr:Q5J7N1:Q5J7N1_MOUSE	0.88	0.95	0.94	0.98	1.0	1.0
tr:Q9D091:Q9D091_MOUSE	0.87	0.96	0.91	0.97	1.0	1.0
tr:Q4FJP3:Q4FJP3_MOUSE	0.85	0.94	0.91	0.97	1.0	1.0
sp:P08646:RAS1_DROME	0.76	0.91	0.81	0.93	1.0	1.0
sp:P22981:LET60_CAEEL	0.75	0.89	0.77	0.92	1.0	1.0
sp:P01119:RAS1_YEAST	0.35	0.51	0.6	0.86	1.0	1.0
sp:P01120:RAS2_YEAST	0.34	0.5	0.61	0.86	1.0	1.0

Ras85D (FBgn0003205) associated phenotypes

RU486 conditional, cell autonomous, cell death defective, cell non-autonomous, cell polarity defective, cell size defective, conditional, decreased cell death, decreased cell number, decreased cell size, developmental rate defective, dominant, endocytosis defective, female semi-sterile, germline clone, heat sensitive, hyperplasia, increased cell death, increased cell growth, increased cell number, increased cell size, large body, learning defective, lethal - all die before end of P-stage, lethal - all die before end of pupal stage, majority die during P-stage, maternal effect, melanotic mass phenotype,

mitotic cell cycle defective, neoplasia, neuroanatomy defective, neurophysiology defective, partially, partially lethal - majority die, short lived, size defective, small body, somatic clone, some die during P-stage, some die during pharate adult stage, some die during pupal stage, temperature conditional, terminal phenotype, tumorigenic

(Information from FlyBase)

Ras85D (UniProt:P08646) annotation
Function: May mediate a signal that determines the fate of photoreceptor cells in the developing compound eye. Ras proteins bind GDP/GTP and possess intrinsic GTPase activity.
Enzyme regulation: Alternates between an inactive form bound to GDP and an active form bound to GTP. Activated by a guanine nucleotide-exchange factor (GEF) and inactivated by a GTPase- activating protein (GAP).
Subcellular location: Cell membrane (PubMed:18503409); Lipid-anchor (PubMed:18503409); Cytoplasmic side (PubMed:18503409). Note=Loss of prenylation causes protein location to the cytoplasm.
Tissue specificity: Expressed in the posterior termini of the embryo, restricted mainly to the embryonic central nervous system, and in the eye imaginal disk. (PubMed:7873789).

(Information from UniProt)

let-60 (WBGene00002335) associated phenotypes
AWA odorant chemotaxis defective, AWC odorant chemotaxis defective, L2 arrest, L3 arrest, antibody staining reduced, antibody staining variant, axon outgrowth variant, bloated, cell differentiation variant, egg laying defective, egg laying imipramine resistant, egg laying variant, germ cell development variant, gonad arm morphology variant, lethal, locomotion variant, male mating defective, mid larval lethal, multivulva, neuron degeneration, oocyte accumulation, oocytes small, organism starvation response variant, protein expression absent, reduced brood size, rod like larval lethal, sterile, unfertilized oocytes laid, vulval cell induction increased, vulvaless

(Information from WormBase)

let-60 (UniProt:P22981) annotation
Function: The level of let-60 controls the switch between vulval and hypodermal cell fates during C.elegans vulval induction. May stimulate the guanine nucleotide exchange factor (GEF) activity of rap-1.
Subunit: Interacts with soc-2. (PubMed:9674433).
Subcellular location: Cell membrane; Lipid-anchor.

(Information from UniProt)

Heat shock protein HSP 90-alpha

Best gene implication in ageing for this target family came from gene Q18688 annotated in UniProt release 2014_02. Annotation GO 8340 (determination of adult lifespan) was Inferred from Genetic Interaction

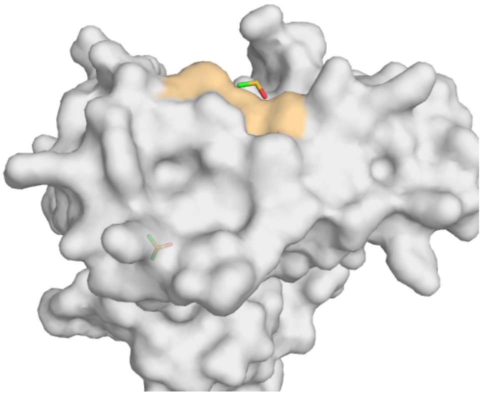

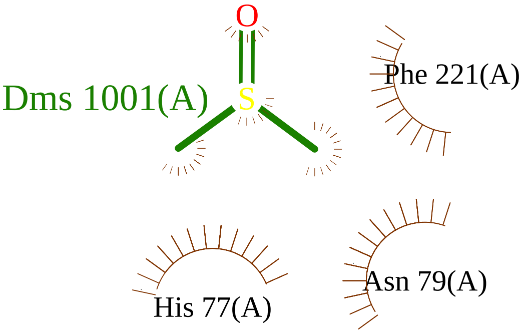

protein	amino acids contacts (binding site)
PDB:2jjc:chainA:P07900	S I N S D A E H N D M A G T T F
PDB:2wi2:chainA:P07900	S I N S D A E H N D M A G T T F
PDB:2xds:chainA:P07900	S I N S D A E H N D M A G T T F
PDB:2xdu:chainA:P07900	S I N S D A E H N D M A G T T F
PDB:3inw:chainA:P07900	S I N S D A E H N D M A G T T F
PDB:3inx:chainA:P07900	S I N S D A E H N D M A G T T F
PDB:3k97:chainA:P07900	S I N S D A E H N D M A G T T F
PDB:3qtf:chainA:P07900	S I N S D A E H N D M A G T T F
tr:K9JA46:K9JA46_HUMAN	S I N S D A E H N D M A G T T F
sp:P07900:HS90A_HUMAN	S I N S D A E H N D M A G T T F
sp:P82995:HS90A_RAT	S I N S D A E H N D M A G T T F
tr:Q8C2A9:Q8C2A9_MOUSE	S I N S D A E H N D M A G T T F
tr:Q3TKB9:Q3TKB9_MOUSE	S I N S D A E H N D M A G T T F
tr:Q3TKG0:Q3TKG0_MOUSE	S I N S D A E H N D M A G T T F
sp:P07901:HS90A_MOUSE	S I N S D A E H N D M A G T T F
tr:Q80Y52:Q80Y52_MOUSE	S I N S D A E H N D M A G T T F
sp:P02828:HSP83_DROME	S I N A D A E Y K D M A G T K L
sp:Q18688:HSP90_CAEEL	S I N A D A E F K D M A G T K V
sp:P02829:HSP82_YEAST	S I N A D A D F R D M A G T Q V

	whole protein		domain-based		contact-based	
protein	ident	simil	ident	simil	ident	simil
PDB:2jjc:chainA:P07900	1.0	1.0	1.0	1.0	1.0	1.0
PDB:2wi2:chainA:P07900	1.0	1.0	1.0	1.0	1.0	1.0
PDB:2xds:chainA:P07900	1.0	1.0	1.0	1.0	1.0	1.0
PDB:2xdu:chainA:P07900	1.0	1.0	1.0	1.0	1.0	1.0
PDB:3inw:chainA:P07900	1.0	1.0	1.0	1.0	1.0	1.0
PDB:3inx:chainA:P07900	1.0	1.0	1.0	1.0	1.0	1.0
PDB:3k97:chainA:P07900	1.0	1.0	1.0	1.0	1.0	1.0
PDB:3qtf:chainA:P07900	1.0	1.0	1.0	1.0	1.0	1.0
tr:K9JA46:K9JA46_HUMAN	1.0	1.0	1.0	1.0	1.0	1.0
sp:P07900:HS90A_HUMAN	1.0	1.0	1.0	1.0	1.0	1.0
sp:P82995:HS90A_RAT	0.99	1.0	1.0	1.0	1.0	1.0
tr:Q8C2A9:Q8C2A9_MOUSE	0.37	0.37	1.0	1.0	1.0	1.0
tr:Q3TKB9:Q3TKB9_MOUSE	0.75	0.76	1.0	1.0	1.0	1.0
tr:Q3TKG0:Q3TKG0_MOUSE	0.75	0.76	1.0	1.0	1.0	1.0
sp:P07901:HS90A_MOUSE	0.99	1.0	1.0	1.0	1.0	1.0
tr:Q80Y52:Q80Y52_MOUSE	0.99	1.0	1.0	1.0	1.0	1.0
sp:P02828:HSP83_DROME	0.77	0.92	0.82	0.91	0.69	0.78
sp:Q18688:HSP90_CAEEL	0.73	0.89	0.73	0.89	0.69	0.76
sp:P02829:HSP82_YEAST	0.6	0.84	0.65	0.85	0.63	0.75

Hsp83 (FBgn0001233) associated phenotypes

cell cycle defective, cell polarity defective, developmental rate defective, female semi-fertile, lethal - all die before end of pupal stage, lethal - all die during third instar larval stage, partially lethal, partially lethal - majority die, short lived

(Information from FlyBase)

Hsp83 (UniProt:P02828) annotation
Function: Molecular chaperone that promotes the maturation, structural maintenance and proper regulation of specific target proteins involved for instance in cell cycle control and signal transduction. Undergoes a functional cycle that is linked to its ATPase activity. This cycle probably induces conformational changes in the client proteins, thereby causing their activation. Interacts dynamically with various co-chaperones that modulate its substrate recognition, ATPase cycle and chaperone function. Together with Hop and piwi, mediates canalization, also known as developmental robustness, likely via epigenetic silencing of existing genetic variants and suppression of transposon-induced new genetic variation. Required for piRNA biogenesis by facilitating loading of piRNAs into PIWI proteins. (PubMed:21186352, PubMed:22902557).
Subunit: Homodimer (By similarity). Forms a complex with Hop and piwi; probably Hop mediates the interaction between piwi and Hsp83. Interacts with shu. (, PubMed:21186352, PubMed:22902557).
Subcellular location: Cytoplasm.
Induction: In contrast to other major heat shock proteins, this one is also expressed at normal growth temperatures. It is also developmentally expressed during oogenesis.
Domain: The TPR repeat-binding motif mediates interaction with TPR repeat-containing proteins.

(Information from UniProt)

daf-21 (WBGene00000915) associated phenotypes
chemotaxis variant, dauer constitutive, dauer recovery variant, larval lethal, lethal, odorant chemosensory response variant, sluggish, sterile

(Information from WormBase)

daf-21 (UniProt:Q18688) annotation
Function: Molecular chaperone that promotes the maturation, structural maintenance and proper regulation of specific target proteins involved for instance in cell cycle control and signal transduction. Undergoes a functional cycle that is linked to its ATPase activity. This cycle probably induces conformational changes in the client proteins, thereby causing their activation. Interacts dynamically with various co-chaperones that modulate its substrate recognition, ATPase cycle and chaperone function. By stabilizing the receptor-type guanylate cyclase daf-11 or another signal transduction component that regulates cGMP levels, plays a role in dauer formation and chemotaxis to non-volatile and volatile attractants detected by AWC sensory neurons (PubMed:10790386, PubMed:7828815). Participates in the control of cell cycle progression at the prophase/metaphase transition in oocyte development by ensuring the activity of wee-1.3 kinase, which negatively regulates cdk-1 through its phosphorylation (PubMed:16466390). Regulates yap-1 nuclear export after heat shock treatment (PubMed:23396260). (PubMed:10790386, PubMed:16466390, PubMed:23396260, PubMed:7828815).
Subunit: Homodimer. (UniProtKB:P08238).
Subcellular location: Cytoplasm, perinuclear region (PubMed:12950278). Note=Perinuclear region of somatic cells.
Tissue specificity: In the embryo comma stage, expression is strongly detected in cells of the head region and less so in other areas. In early larvae, expressed in postembryonic germ cells derived from Z2 and Z3 cells and the head region, in both hermaphrodites and males. Under heat stress conditions, larval expression is not only detected in germ cells, but also all over the body. In adult hermaphrodites, expression is localized uniquely in the germ cells. (PubMed:12950278).
Developmental stage: Highly expressed throughout development. (PubMed:10790386).
Domain: The TPR repeat-binding motif mediates interaction with TPR repeat-containing proteins.

(Information from UniProt)

Peroxiredoxin-5, mitochondrial

Best gene implication in ageing for this target family came from gene Q960M4 annotated in UniProt release 2014_02. Annotation GO 8340 (determination of adult lifespan) was Inferred from Mutant Phenotype

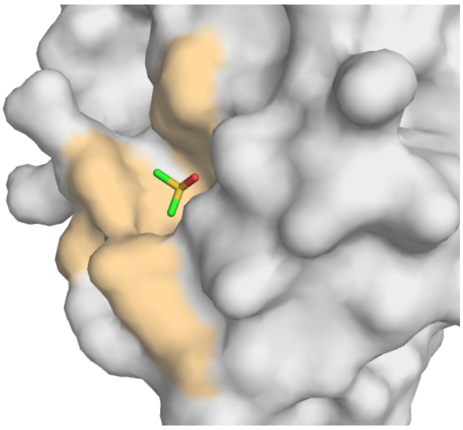

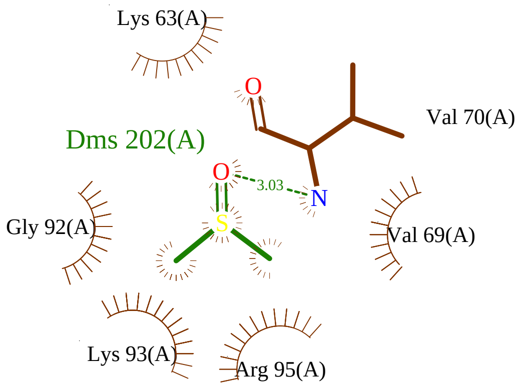

protein	amino acids contacts (binding site)									
PDB:4k7i:chainA:P30044	F	S	K	V	V	W	H	G	K	R
PDB:4k7o:chainA:P30044	F	S	K	V	V	W	H	G	K	R
sp:P30044:PRDX5_HUMAN	F	S	K	V	V	W	H	G	K	R
tr:D3ZEN5:D3ZEN5_RAT	F	S	K	V	V	W	H	G	K	Q
sp:Q9R063:PRDX5_RAT	F	S	K	V	V	W	H	G	K	Q
tr:Q3UWS9:Q3UWS9_MOUSE	F	S	K	V	V	W	H	G	K	R
sp:P99029:PRDX5_MOUSE	F	S	K	V	V	W	H	G	K	R
tr:H3BJQ7:H3BJQ7_MOUSE	F	S	K	V	V	W	H	G	K	R
tr:Q960M4:Q960M4_DROME	F	S	K	E	I	W	H	G	K	R
sp:P38013:AHP1_YEAST	F	T	V	Q	V	W	L	T	H	K

	whole protein		domain-based		contact-based	
protein	ident	simil	ident	simil	ident	simil
PDB:4k7i:chainA:P30044	0.99	1.0	0.99	1.0	1.0	1.0
PDB:4k7o:chainA:P30044	0.99	1.0	0.99	1.0	1.0	1.0
sp:P30044:PRDX5_HUMAN	1.0	1.0	1.0	1.0	1.0	1.0
tr:D3ZEN5:D3ZEN5_RAT	0.66	0.73	0.84	0.93	0.9	0.96
sp:Q9R063:PRDX5_RAT	0.78	0.91	0.89	0.97	0.9	0.96
tr:Q3UWS9:Q3UWS9_MOUSE	0.76	0.87	0.89	0.97	1.0	1.0
sp:P99029:PRDX5_MOUSE	0.77	0.9	0.89	0.97	1.0	1.0
tr:H3BJQ7:H3BJQ7_MOUSE	0.66	0.78	0.85	0.95	1.0	1.0
tr:Q960M4:Q960M4_DROME	0.46	0.73	0.57	0.84	0.8	0.86
sp:P38013:AHP1_YEAST	0.19	0.55	0.23	0.64	0.3	0.62

Prx5 (FBgn0038570) associated phenotypes

chemical resistant, chemical sensitive, immune response defective, increased cell death, long lived, parental effect, short lived, stress response defective

(Information from FlyBase)

Glycogen synthase kinase-3 beta

Best gene implication in ageing for this target family came from gene P38615 via mapping the annotation from Ensembl YMR139W via mapping the annotation from EntrezGene 855170 via mapping the annotation from GenAgeModels 1320 annotated in GenAge release 17.

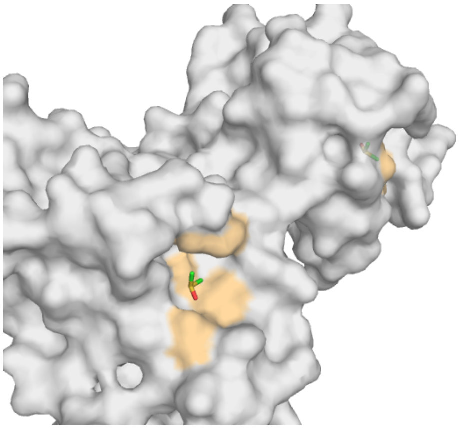

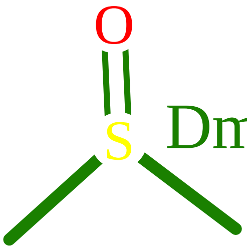

Dms 1385(A)

protein	amino acids contacts (binding site)
PDB:4acc:chainA:P49841	Y T Y K N V Y Y P
tr:Q6FI27:Q6FI27_HUMAN	Y T Y K N V Y Y P
sp:P49841:GSK3B_HUMAN	Y T Y K N V Y Y P
sp:P18266:GSK3B_RAT	Y T Y K N V Y Y P
tr:Q5KU03:Q5KU03_MOUSE	Y T Y K N V Y Y P
sp:Q9WV60:GSK3B_MOUSE	Y T Y K N V Y Y P
tr:E9QAQ5:E9QAQ5_MOUSE	Y T Y K N V Y Y P
tr:B6IDN4:B6IDN4_DROME	Y T F K N V Y Y P
sp:P18431:SGG_DROME	Y T F K N V Y Y P
tr:A4V3W2:A4V3W2_DROME	Y T F K N V Y Y P
tr:A4V3W1:A4V3W1_DROME	Y T F K N V Y Y P
tr:Q0KHW6:Q0KHW6_DROME	Y T F K N V Y Y P
tr:E6PBX9:E6PBX9_DROME	Y T F K N V Y Y P
tr:H5V852:H5V852_DROME	Y T F K N V Y Y P
sp:Q9U2Q9:GSK3_CAEEL	Y Q F K N V Y Y P
sp:P38615:RIM11_YEAST	F T F K N R F Y P
sp:P50873:MRK1_YEAST	Y T V K N R F Y P

	whole protein		domain-based		contact-based	
protein	ident	simil	ident	simil	ident	simil
PDB:4acc:chainA:P49841	1.0	1.0	1.0	1.0	1.0	1.0
tr:Q6FI27:Q6FI27_HUMAN	1.0	1.0	1.0	1.0	1.0	1.0
sp:P49841:GSK3B_HUMAN	1.0	1.0	1.0	1.0	1.0	1.0
sp:P18266:GSK3B_RAT	0.99	1.0	1.0	1.0	1.0	1.0
tr:Q5KU03:Q5KU03_MOUSE	0.99	1.0	1.0	1.0	1.0	1.0
sp:Q9WV60:GSK3B_MOUSE	0.99	1.0	1.0	1.0	1.0	1.0
tr:E9QAQ5:E9QAQ5_MOUSE	0.96	0.97	0.96	0.96	1.0	1.0
tr:B6IDN4:B6IDN4_DROME	0.58	0.71	0.85	0.96	0.89	0.97
sp:P18431:SGG_DROME	0.58	0.73	0.85	0.96	0.89	0.97
tr:A4V3W2:A4V3W2_DROME	0.58	0.73	0.85	0.96	0.89	0.97
tr:A4V3W1:A4V3W1_DROME	0.52	0.65	0.85	0.96	0.89	0.97
tr:Q0KHW6:Q0KHW6_DROME	0.49	0.63	0.85	0.96	0.89	0.97
tr:E6PBX9:E6PBX9_DROME	0.18	0.25	0.52	0.65	0.89	0.97
tr:H5V852:H5V852_DROME	0.28	0.35	0.85	0.96	0.89	0.97
sp:Q9U2Q9:GSK3_CAEEL	0.6	0.76	0.81	0.95	0.78	0.93
sp:P38615:RIM11_YEAST	0.41	0.67	0.57	0.86	0.56	0.81
sp:P50873:MRK1_YEAST	0.31	0.56	0.53	0.84	0.67	0.8

sgg (FBgn0003371) associated phenotypes

cell non-autonomous, cell polarity defective, chemical sensitive, circadian rhythm defective, developmental rate defective, germline clone, heat sensitive, lethal - all die before end of P-stage, lethal - all die before end of pupal stage, maternal effect, meiotic cell cycle defective, mitotic cell cycle defective, neuroanatomy defective, neurophysiology defective, non-rescuable maternal effect, planar polarity defective, rescuable maternal effect, short lived, smell perception defective, somatic clone, some die during pharate adult stage, some die during pupal stage, some die during third instar

larval stage

(Information from FlyBase)

sgg (UniProt:P18431) annotation

Function: Required for several developmental events such as syncytial blastoderm formation and embryonic segmentation. Is involved in transcriptional regulation. Required for arm phosphorylation. Wg signaling operates by inactivating the sgg repression of en autoactivation. Negatively controls the neuromuscular junction (NMJ) growth in presynaptic motoneurons. Plays a role in the regulation of microtubule dynamics and actin cytoskeleton during embryogenesis. Required for phosphorylation of sra in activated eggs. Essential for completion of meiosis, possibly by triggering calcineurin activation via sra phosphorylation. Phosphorylates microtubule-associated protein futsch in axons. (PubMed:15269269, PubMed:16570248, PubMed:16949836, PubMed:2113617, PubMed:2118107, PubMed:22421435, PubMed:7529201, PubMed:8467811).

Subunit: Interacts with cos. (PubMed:1335365,PubMed:15691767).

Subcellular location: Cytoplasm. Nucleus. Cytoplasm, cytoskeleton, microtubule organizing center, centrosome. Cytoplasm, cell cortex. Cell junction, synapse. Cell projection, axon. Note=In syncytial embryos, detected at the centrosomes throughout the cell cycle, and in the mitotic spindle and pseudocleavage furrows invaginating from the cell cortex during mitosis. Concentrated at the growing end of membranes during the cellularization process. After cellularization, localized to the centrosomes during mitosis and to the nucleus at the end of telophase. Enriched in the presynaptic side of the neuromuscular junction, with some signal detected also in axonal branches.

Tissue specificity: Expressed in ovaries and activated eggs (at protein level). Expression is over all the embryo at all stages, no local accumulation is observed. (PubMed:2118107, PubMed:22421435, PubMed:8467811).

Developmental stage: Isoform SGG46 is expressed at low levels in 12-24 hours embryos. Isoform Zygotic and isoform SGG39 are expressed in 12-24 hours embryos and present throughout the larval, pupal and adult stages (at protein level). Isoform Zygotic is expressed maternally and zygotically but reduced throughout later embryonic development. Expression persists throughout larval stages. (PubMed:2118107, PubMed:8467811).

Disruption phenotype: Mutants display an overdeveloped neuromuscular junction (NMJ), with the number of boutons greatly increased. (PubMed:15269269).

(Information from UniProt)

gsk-3 (WBGene00001746) associated phenotypes

axon morphology variant, larval lethal, lethal, locomotion variant, sterile

(Information from WormBase)

gsk-3 (UniProt:Q9U2Q9) annotation

Function: Phosphorylates oma-1, a regulator of the oocyte-to- embryo transition, enabling its degradation. Phosphorylates skn-1, preventing it from accumulating in nuclei and thus inhibiting phase II gene expression in the oxidative stress defense. Involved in mesendoderm specification and mitotic spindle orientation in EMS blastomeres. Thought to be a branch point in these processes as proteins downstream are not required. Negatively regulates Wnt signaling in vulval precursor cells and acts as a Wnt-independent repressor of med-1 and med-2 in the C lineage inhibiting mesoderm development. Required for normal lifespan and LiCl-induced lifespan extension. (PubMed:10444600, PubMed:11463373, PubMed:15572126, PubMed:16251270, PubMed:16289132, PubMed:16343905, PubMed:16930586, PubMed:17959600).

Subunit: Monomer (By similarity). Interacts with axl-1. (UniProtKB:Q9WV60, PubMed:17601533).

Disruption phenotype: Worms exhibit defects in endoderm specification and mitotic spindle alignment. Mutants show reduced degradation of oma-1 and have shortened lifespan. (PubMed:10444600).

(Information from UniProt)

Cyclin-dependent kinase 6

Best gene implication in ageing for this target family came from gene Q99MD0 via mapping the annotation from RGD 621121 annotated in RGD 2014-03-11. Annotation GO subterm of 7568 (ageing) was Inferred from Sequence Orthology

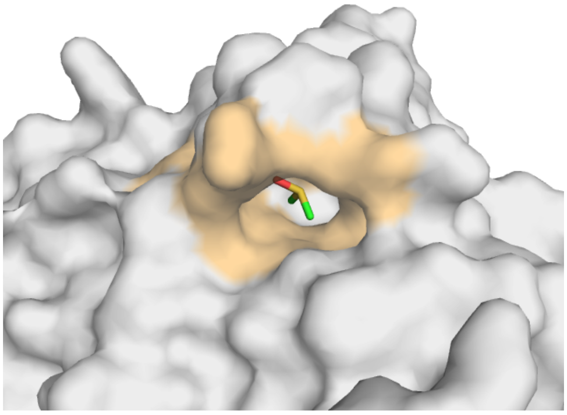

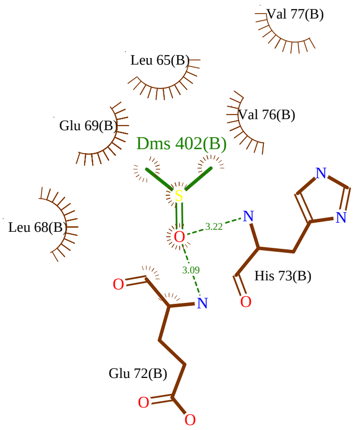

protein	amino acids contacts (binding site)							
PDB:2euf:chainB:Q00534	L	L	E	E	H	V	V	R
PDB:2f2c:chainB:Q00534	L	L	E	E	H	V	V	R
sp:Q00534:CDK6_HUMAN	L	L	E	E	H	V	V	R
tr:Q99MD0:Q99MD0_RAT	L	L	E	E	H	V	V	R
tr:F1MA87:F1MA87_RAT	L	L	E	E	H	V	V	R
sp:Q64261:CDK6_MOUSE	L	L	E	E	H	V	V	R
tr:Q0VBK8:Q0VBK8_MOUSE	L	L	E	E	H	V	V	R
tr:Q94877:Q94877_DROME	L	L	N	N	H	I	V	K
tr:Q7K306:Q7K306_DROME	L	L	N	N	H	I	V	K
tr:Q20643:Q20643_CAEEL	L	H	E	Q	R	I	V	E
sp:P00546:CDK1_YEAST	L	L	-	K	D	I	V	R

	whole protein		domain-based		contact-based	
protein	ident	simil	ident	simil	ident	simil
PDB:2euf:chainB:Q00534	1.0	1.0	1.0	1.0	1.0	1.0
PDB:2f2c:chainB:Q00534	1.0	1.0	1.0	1.0	1.0	1.0
sp:Q00534:CDK6_HUMAN	1.0	1.0	1.0	1.0	1.0	1.0
tr:Q99MD0:Q99MD0_RAT	0.34	0.34	0.29	0.29	1.0	1.0
tr:F1MA87:F1MA87_RAT	0.97	0.99	0.98	0.99	1.0	1.0
sp:Q64261:CDK6_MOUSE	0.96	0.99	0.98	0.99	1.0	1.0
tr:Q0VBK8:Q0VBK8_MOUSE	0.96	0.99	0.98	0.99	1.0	1.0
tr:Q94877:Q94877_DROME	0.4	0.74	0.44	0.82	0.5	0.84
tr:Q7K306:Q7K306_DROME	0.4	0.74	0.44	0.82	0.5	0.84
tr:Q20643:Q20643_CAEEL	0.22	0.62	0.26	0.69	0.38	0.75
sp:P00546:CDK1_YEAST	0.41	0.71	0.44	0.76	0.5	0.56

Cdk4 (FBgn0016131) associated phenotypes

decreased cell number, increased cell size, maternal effect, poor, radiation sensitive, size defective, small body, somatic clone, sterile

(Information from FlyBase)

Tyrosine-protein kinase BTK

Best gene implication in ageing for this target family came from gene P08630 annotated in UniProt release 2014_02. Annotation GO 8340 (determination of adult lifespan) was Inferred from Mutant Phenotype

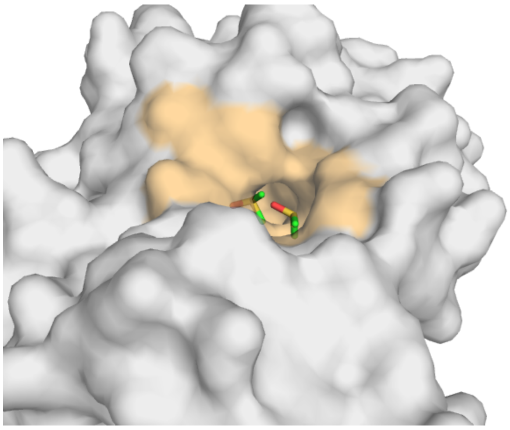

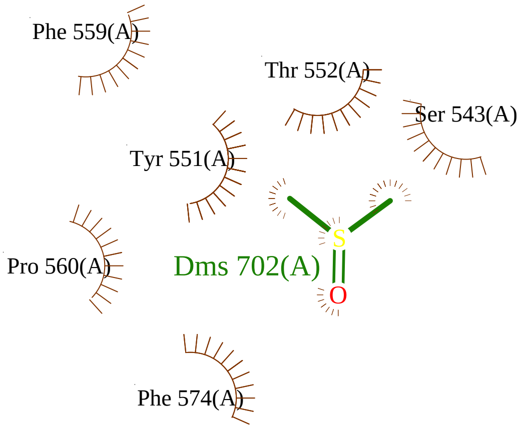

protein	amino acids contacts (binding site)
PDB:4ot5:chainA:Q06187	R S Y T F P W S F S
PDB:4otq:chainA:Q06187	R S Y T F P W S F S
PDB:4otr:chainA:Q06187	R S Y T F P W S F S
tr:Q3MS96:Q3MS96_HUMAN	R S Y T F P W S F S
sp:Q06187:BTK_HUMAN	R S Y T F P W S F S
tr:E9PTF7:E9PTF7_RAT	R R Y T F P W S F S
sp:P35991:BTK_MOUSE	R S Y T F P W S F S
sp:P08630:BTKL_DROME	R A Y T F P W A F S

	whole protein		domain-based		contact-based	
protein	ident	simil	ident	simil	ident	simil
PDB:4ot5:chainA:Q06187	0.98	0.99	0.98	0.99	1.0	1.0
PDB:4otq:chainA:Q06187	0.98	0.99	0.98	0.99	1.0	1.0
PDB:4otr:chainA:Q06187	0.98	0.99	0.98	0.99	1.0	1.0
tr:Q3MS96:Q3MS96_HUMAN	0.48	0.48	0.95	0.95	1.0	1.0
sp:Q06187:BTK_HUMAN	1.0	1.0	1.0	1.0	1.0	1.0
tr:E9PTF7:E9PTF7_RAT	0.93	0.97	0.9	0.96	0.9	0.9
sp:P35991:BTK_MOUSE	0.98	1.0	0.99	1.0	1.0	1.0
sp:P08630:BTKL_DROME	0.34	0.62	0.56	0.86	0.8	0.82

Btk29A (FBgn0003502) associated phenotypes

cell shape defective, lethal - all die before end of first instar larval stage, mating defective, partially, short lived, size defective, some die during first instar larval stage

(Information from FlyBase)

Btk29A (UniProt:P08630) annotation

Function: Required for proper ring canal development. Also required for the development of male genitalia and for adult survival. (PubMed:10330180, PubMed:9655810).
Cofactor: Zn(2+)Note=Binds 1 zinc ion per subunit. ;
Subcellular location: Note=Ring canals.
Tissue specificity: Ring canals in the egg chambers and imaginal disks of third-instar larvae. (PubMed:10330180, PubMed:3110602, PubMed:9655810).
Developmental stage: Expressed both maternally and zygotically. Predominantly in early to middle embryogenesis, in larvae and adult females. (PubMed:10330180, PubMed:3110602).
Disruption phenotype: Flies exhibit shortened copulatory duration (due to incomplete fusion of the left and right halves of the apodeme that holds the penis during copulation) and reduced adult-stage life span. (PubMed:10330180).

(Information from UniProt)

Histone acetyltransferase p300

Best gene implication in ageing for this target family came from gene P34545 via mapping the annotation from WormBase WBGene00000366 annotated in WormBase 2014-03-11. Annotation GO 8340 (determination of adult lifespan) was Inferred from Mutant Phenotype

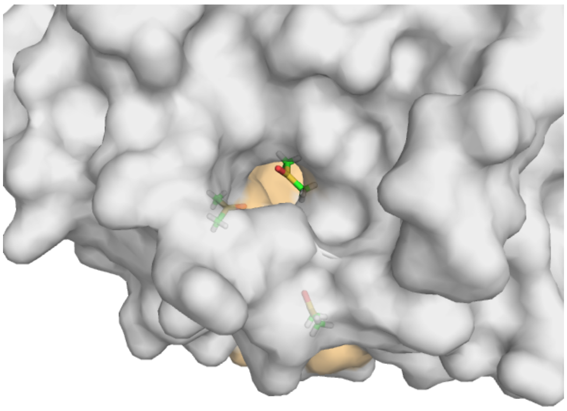

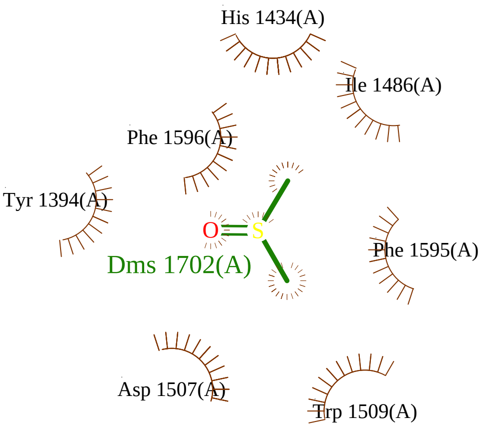

protein	amino acids contacts (binding site)
PDB:4pzr:chainA:Q09472	Y H I W K D I D W H E V F F
PDB:4pzs:chainA:Q09472	Y H I W K D I D W H E V F F
PDB:4pzt:chainA:Q09472	Y H I W K D I D W H E V F F
sp:Q09472:EP300_HUMAN	Y H I W K D I D W H E V F F
sp:Q92793:CBP_HUMAN	Y H I W K D I D W H E V F F
tr:Q91XT0:Q91XT0_RAT	- - - - - - - - - - - - - -
sp:B2RWS6:EP300_MOUSE	Y H I W K D I D W H E V F F
tr:O01368:O01368_DROME	Y H I W K D I D W H E V F F
tr:Q9W321:Q9W321_DROME	Y H I W K D I D W H E V F F
sp:P34545:CBP1_CAEEL	Y H I W K D I D W H E V F F
sp:P35817:BDF1_YEAST	- G L D Q - - H G - - - - -

	whole protein		domain-based		contact-based	
protein	ident	simil	ident	simil	ident	simil
PDB:4pzr:chainA:Q09472	1.0	1.0	1.0	1.0	1.0	1.0
PDB:4pzs:chainA:Q09472	1.0	1.0	1.0	1.0	1.0	1.0
PDB:4pzt:chainA:Q09472	1.0	1.0	1.0	1.0	1.0	1.0
sp:Q09472:EP300_HUMAN	1.0	1.0	1.0	1.0	1.0	1.0
sp:Q92793:CBP_HUMAN	0.56	0.81	0.9	0.97	1.0	1.0
tr:Q91XT0:Q91XT0_RAT	0.14	0.14	0.0	0.0	0.0	0.0
sp:B2RWS6:EP300_MOUSE	0.94	0.98	0.99	1.0	1.0	1.0
tr:O01368:O01368_DROME	0.26	0.54	0.64	0.86	1.0	1.0
tr:Q9W321:Q9W321_DROME	0.26	0.56	0.64	0.86	1.0	1.0
sp:P34545:CBP1_CAEEL	0.27	0.56	0.57	0.85	1.0	1.0
sp:P35817:BDF1_YEAST	0.03	0.16	0.04	0.35	0.0	0.0

nej (FBgn0261617) associated phenotypes

cell migration defective, chemical sensitive, developmental rate defective, dominant, gravitaxis defective, heat sensitive, hypoactive, lethal - all die before end of P-stage, lethal - all die before end of prepupal stage, lethal - all die before end of pupal stage, mitotic cell cycle defective, neuroanatomy defective, neurophysiology defective, partially lethal - majority die, partially lethal - majority live, somatic clone, some die during P-stage, some die during pharate adult stage, some die during prepupal stage, some die during pupal stage

(Information from FlyBase)

cbp-1 (WBGene00000366) associated phenotypes

bag of worms, embryonic lethal, larval lethal, protruding vulva, sick, slow growth, sterile, vulvaless

(Information from WormBase)

cbp-1 (UniProt:P34545) annotation

Function: Acetyltransferase enzyme. Acetylates histones, giving a specific tag for transcriptional activation (By similarity). May prevent DNA damage-induced apoptosis by inhibiting cep-1-dependent transcription activation of the programmed cell death activator egl-1 (PubMed:19521535). (UniProtKB:Q92793, PubMed:19521535).

Subunit: Interacts (via N-terminus domain and HAT domain) with prmt-5; the interaction results in methylation of cbp-1 (PubMed:19521535). Interacts (via HAT domain) with cep-1; cep-1 transcriptional activity may be inhibited by interaction with methylated cbp-1 (PubMed:19521535). Component of a complex that contains prmt-5 and cbp-1 (PubMed:19521535). (PubMed:19521535, PubMed:19521535).

Subcellular location: Nucleus (UniProtKB:Q92793).

Ptm: Methylation by prmt-5 may repress the capacity of cbp-1 to enhance cep-1-dependent transcription of egl-1. (PubMed:19521535).

(Information from UniProt)

Mitogen-activated protein kinase 14

Best gene implication in ageing for this target family came from gene O62618 annotated in UniProt release 2014_02. Annotation GO 8340 (determination of adult lifespan) was Inferred from Mutant Phenotype

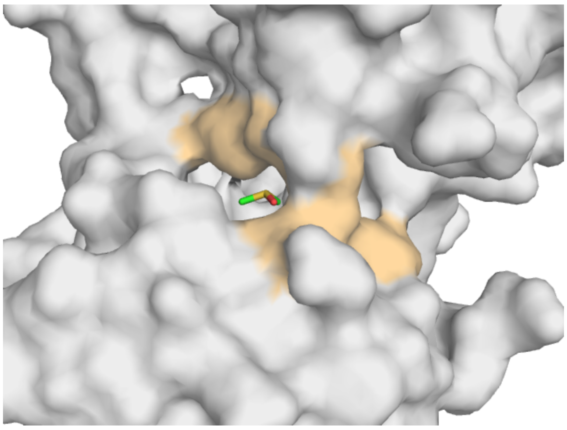

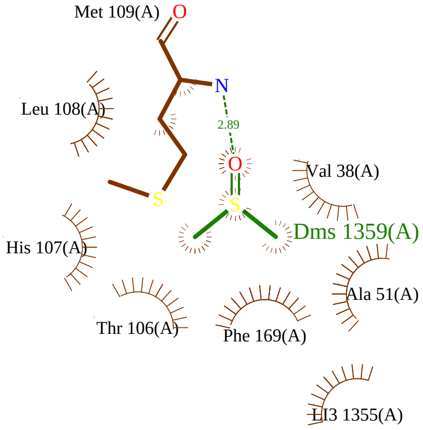

protein	amino acids contacts (binding site)
PDB:1wbv:chainA:Q16539	V A T H L M F
tr:L7RSM2:L7RSM2_HUMAN	V A T H L M F
sp:Q16539:MK14_HUMAN	V A T H L M F
tr:G3V617:G3V617_RAT	V A T H L M F
tr:Q56A33:Q56A33_RAT	V A T H L M F
tr:B2KF34:B2KF34_MOUSE	V A T H L M F
tr:B2KF35:B2KF35_MOUSE	V A T H L M F
sp:P47811:MK14_MOUSE	V A T H L M F
tr:Q5U421:Q5U421_MOUSE	V A T H L M F
sp:P83100:MK14C_DROME	V A T H L M F
sp:O62618:MK14A_DROME	V A T H L M F
tr:E1JIV6:E1JIV6_DROME	V A T H L M F
sp:O61443:MK14B_DROME	V A T H L M F
sp:Q17446:PMK1_CAEEL	V A S M L M F
sp:Q8MXI4:PMK2_CAEEL	V A S V L M F
sp:P32485:HOG1_YEAST	V A T E L Q F

protein	whole protein ident	whole protein simil	domain-based ident	domain-based simil	contact-based ident	contact-based simil
PDB:1wbv:chainA:Q16539	1.0	1.0	1.0	1.0	1.0	1.0
tr:L7RSM2:L7RSM2_HUMAN	0.96	0.99	0.95	0.99	1.0	1.0
sp:Q16539:MK14_HUMAN	1.0	1.0	1.0	1.0	1.0	1.0
tr:G3V617:G3V617_RAT	0.99	1.0	0.99	1.0	1.0	1.0
tr:Q56A33:Q56A33_RAT	0.95	0.99	0.95	0.99	1.0	1.0
tr:B2KF34:B2KF34_MOUSE	0.56	0.56	0.62	0.62	1.0	1.0
tr:B2KF35:B2KF35_MOUSE	0.7	0.71	0.8	0.8	1.0	1.0
sp:P47811:MK14_MOUSE	0.99	1.0	0.99	1.0	1.0	1.0
tr:Q5U421:Q5U421_MOUSE	0.96	0.99	0.95	0.99	1.0	1.0
sp:P83100:MK14C_DROME	0.44	0.79	0.46	0.83	1.0	1.0
sp:O62618:MK14A_DROME	0.67	0.88	0.74	0.91	1.0	1.0
tr:E1JIV6:E1JIV6_DROME	0.67	0.88	0.74	0.91	1.0	1.0
sp:O61443:MK14B_DROME	0.68	0.89	0.76	0.92	1.0	1.0
sp:Q17446:PMK1_CAEEL	0.61	0.84	0.71	0.92	0.71	0.78
sp:Q8MXI4:PMK2_CAEEL	0.49	0.74	0.59	0.84	0.71	0.78
sp:P32485:HOG1_YEAST	0.41	0.68	0.54	0.85	0.71	0.78

p38c (UniProt:P83100) annotation
Function: Kinase involved in a signal transduction pathway.
Cofactor:Mg(2+)
Ptm: The phosphorylation on Thr-177 activates the enzyme (By similarity). A conserved Tyr, which must also be phosphorylated to activate the enzyme in closely related sequences, is replaced by His-179 in this sequence.

(Information from UniProt)

Mpk2 (FBgn0015765) associated phenotypes
heat stress response defective, immune response defective, non-enhancer of variegation, non-suppressor of variegation, nutrition conditional, osmotic stress response defective, oxidative stress response defective, partially lethal - majority die, reduced, short lived, starvation stress response defective, stress response defective

(Information from FlyBase)

Mpk2 (UniProt:O62618) annotation
Function: Kinase involved in a signal transduction pathway. May down-regulate insect immunity gene expression after prolonged infection. (PubMed:9417090, PubMed:9584193).
Cofactor:Mg(2+)
Enzyme regulation: Activated by threonine and tyrosine phosphorylation by Mkk3 in response to environmental stress. (PubMed:9417090, PubMed:9584193).
Subcellular location: Nucleus (PubMed:9417090).
Developmental stage: Expressed both maternally and zygotically. Levels are highest at the preblastoderm stage but low levels are present throughout development. (PubMed:9584193).
Domain: The TXY motif contains the threonine and tyrosine residues whose phosphorylation activates the MAP kinases.
Ptm: Dually phosphorylated on Thr-184 and Tyr-186, which activates the enzyme. (PubMed:9417090).

(Information from UniProt)

p38b (FBgn0024846) associated phenotypes
RU486 conditional, circadian rhythm defective, decreased cell size, developmental rate defective, drug conditional, heat stress response defective, immune response defective, locomotor behavior defective, long lived, melanotic mass phenotype, neuroanatomy defective, nutrition conditional, osmotic stress response defective, oxidative stress response defective, partially lethal - majority die, progressive, short lived, small body, starvation stress response defective

(Information from FlyBase)

p38b (UniProt:O61443) annotation
Function: Kinase involved in dpp signal transduction pathway in the process of wing morphogenesis when the levels of dpp are enhanced or inhibited. May down-regulate insect immunity gene expression after prolonged infection. (PubMed:10022918, PubMed:9584193).
Cofactor:Mg(2+)
Enzyme regulation: Activated by threonine and tyrosine phosphorylation by Mkk3. (PubMed:9584193).
Subcellular location: Nucleus.
Tissue specificity: At mid-embryogenesis, highest expression is seen in developing anterior and posterior midguts. Almost ubiquitous expression throughout all development.
Developmental stage: Expressed both maternally and zygotically in the embryo, expression seen in all developmental stages.
Domain: The TXY motif contains the threonine and tyrosine residues whose phosphorylation activates the MAP kinases.
Ptm: Dually phosphorylated on Thr-183 and Tyr-185, which activates the enzyme.
(Information from UniProt)

pmk-1 (WBGene00004055) associated phenotypes
cell stress response variant, drug induced gene expression variant, gene expression level reduced, lethal, life span variant, organism oxidative stress response hypersensitive, pathogen induced gene expression variant, pathogen susceptibility increased, sterile
(Information from WormBase)

pmk-1 (UniProt:Q17446) annotation
Function: Responds to activation by environmental stress and pro- inflammatory cytokines by phosphorylating downstream targets. Functions downstream of the MAPKK sek-1 and the MAPKKK nsy-1 as the MAP kinase required for pathogen resistance. (PubMed:11703092, PubMed:12142542).
Cofactor: Mg(2+) Evidence=(PubMed:11703092);
Enzyme regulation: Activated by phosphorylation on threonine and tyrosine. Inhibited by pyridinyl-imidazole related compounds. (PubMed:11703092).
Domain: The TXY motif contains the threonine and tyrosine residues whose phosphorylation activates the MAP kinases.
Ptm: Dually phosphorylated on Thr-191 and Tyr-193, which activates the enzyme.
(Information from UniProt)

pmk-2 (UniProt:Q8MXI4) annotation
Function: Responds to activation by environmental stress and pro- inflammatory cytokines by phosphorylating downstream targets. (PubMed:11703092).
Cofactor: Mg(2+) Evidence=(PubMed:11703092);
Enzyme regulation: Activated by phosphorylation on threonine and tyrosine. Inhibited by pyridinyl-imidazole related compounds. (PubMed:11703092).
Subcellular location: Cytoplasm (PubMed:11703092).
Domain: The TXY motif contains the threonine and tyrosine residues whose phosphorylation activates the MAP kinases.
Ptm: Dually phosphorylated on Thr-222 and Tyr-224, which activates the enzyme.
(Information from UniProt)

CREB-binding protein

Best gene implication in ageing for this target family came from gene Q92793 via mapping the annotation from Ensembl ENSG00000005339 via mapping the annotation from EntrezGene 1387 via mapping the annotation from GenAgeHuman 0064 annotated in GenAge release 17.

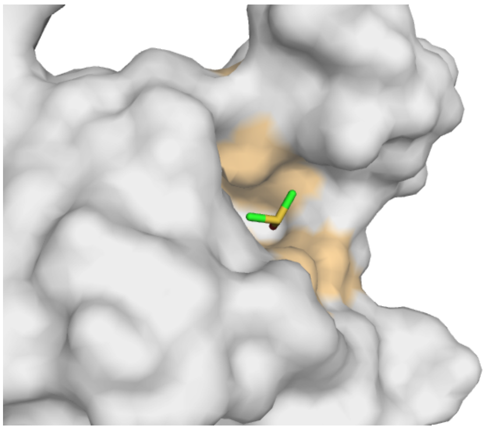

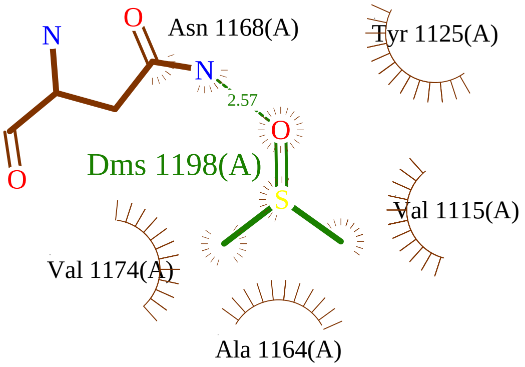

protein	amino acids contacts (binding site)
PDB:3p1e:chainA:Q92793	V Y A N V
sp:Q09472:EP300_HUMAN	V Y A N V
sp:Q92793:CBP_HUMAN	V Y A N V
tr:F1M9G7:F1M9G7_RAT	V Y A N V
tr:Q8QZV8:Q8QZV8_MOUSE	V Y A N V
tr:F8VPR5:F8VPR5_MOUSE	V Y A N V
tr:Q8T3Z6:Q8T3Z6_DROME	V Y C N V
tr:Q058W2:Q058W2_DROME	V Y C N V
tr:Q9VCG6:Q9VCG6_DROME	V Y C N V
tr:Q8T3Z8:Q8T3Z8_DROME	V Y C N V
tr:Q4V5A7:Q4V5A7_DROME	V Y C N V
tr:O17681:O17681_CAEEL	- - - - -
tr:Q9TZB8:Q9TZB8_CAEEL	- - - - -
sp:Q07442:BDF2_YEAST	V Y C N V
sp:P35817:BDF1_YEAST	V Y C N V

	whole protein		domain-based		contact-based	
protein	ident	simil	ident	simil	ident	simil
PDB:3p1e:chainA:Q92793	0.97	0.99	1.0	1.0	1.0	1.0
sp:Q09472:EP300_HUMAN	0.59	0.81	0.86	0.96	1.0	1.0
sp:Q92793:CBP_HUMAN	1.0	1.0	1.0	1.0	1.0	1.0
tr:F1M9G7:F1M9G7_RAT	0.96	0.99	0.98	0.99	1.0	1.0
tr:Q8QZV8:Q8QZV8_MOUSE	0.95	0.98	0.98	1.0	1.0	1.0
tr:F8VPR5:F8VPR5_MOUSE	0.96	0.99	0.98	1.0	1.0	1.0
tr:Q8T3Z6:Q8T3Z6_DROME	0.02	0.06	0.29	0.64	0.8	0.55
tr:Q058W2:Q058W2_DROME	0.02	0.09	0.28	0.63	0.8	0.55
tr:Q9VCG6:Q9VCG6_DROME	0.03	0.12	0.29	0.64	0.8	0.55
tr:Q8T3Z8:Q8T3Z8_DROME	0.02	0.14	0.19	0.7	0.8	0.55
tr:Q4V5A7:Q4V5A7_DROME	0.02	0.13	0.19	0.7	0.8	0.55
tr:O17681:O17681_CAEEL	0.01	0.04	0.0	0.0	0.0	0.0
tr:Q9TZB8:Q9TZB8_CAEEL	0.03	0.07	0.0	0.0	0.0	0.0
sp:Q07442:BDF2_YEAST	0.03	0.15	0.29	0.7	0.8	0.55
sp:P35817:BDF1_YEAST	0.03	0.16	0.29	0.71	0.8	0.55

tbrd-1 (FBgn0039124) associated phenotypes

male fertile

(Information from FlyBase)

Collagen type IV alpha-3-binding protein

Best gene implication in ageing for this target family came from gene Q9Y128 via mapping the annotation from Ensembl FBgn0027569 via mapping the annotation from EntrezGene 38928 via mapping the annotation from GenAgeModels 0117 annotated in GenAge release 17.

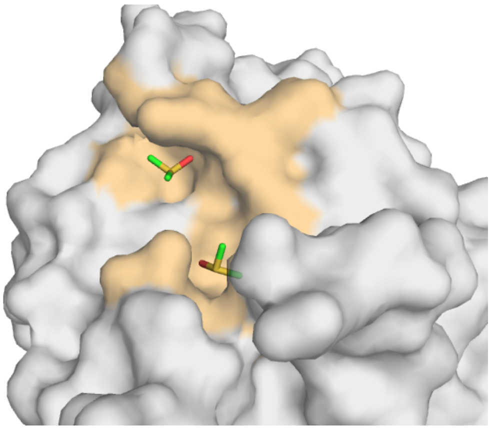

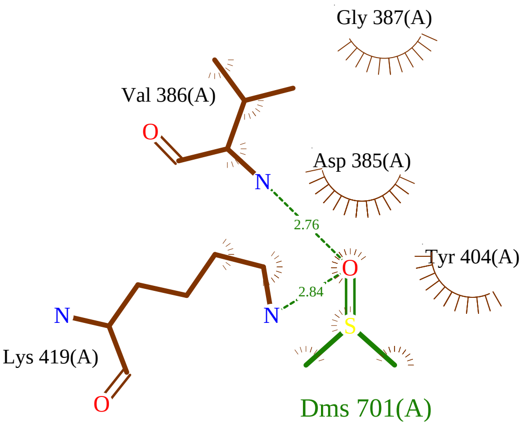

protein	amino acids contacts (binding site)
PDB:2e3o:chainA:Q9Y5P4	L Q D V G Y K I Q K I T
PDB:2e3q:chainA:Q9Y5P4	L Q D V G Y K I Q K I T
sp:Q9Y5P4:C43BP_HUMAN	L Q D V G Y K I Q K I T
tr:D4ACN6:D4ACN6_RAT	L Q D V G Y K I Q K I T
sp:Q9EQG9:C43BP_MOUSE	L Q D V G Y K I Q K I T
tr:Q9Y128:Q9Y128_DROME	R E G V G Y K A Q K I T
tr:Q93569:Q93569_CAEEL	L A - - G Y K I E N I I

	whole protein		domain-based		contact-based	
protein	ident	simil	ident	simil	ident	simil
PDB:2e3o:chainA:Q9Y5P4	0.99	1.0	0.99	1.0	1.0	1.0
PDB:2e3q:chainA:Q9Y5P4	0.98	0.99	0.99	1.0	1.0	1.0
sp:Q9Y5P4:C43BP_HUMAN	1.0	1.0	1.0	1.0	1.0	1.0
tr:D4ACN6:D4ACN6_RAT	0.96	0.99	0.98	1.0	1.0	1.0
sp:Q9EQG9:C43BP_MOUSE	0.97	0.99	0.98	1.0	1.0	1.0
tr:Q9Y128:Q9Y128_DROME	0.42	0.74	0.47	0.82	0.67	0.75
tr:Q93569:Q93569_CAEEL	0.25	0.63	0.37	0.79	0.5	0.43

cert (FBgn0027569) associated phenotypes

heat stress response defective, locomotor behavior defective, short lived, stress response defective

(Information from FlyBase)
